# Celebrating More Than a Century of Research on Antibodies: Affirmation Through Negation via Complex Formation

**DOI:** 10.20411/pai.v2i1.198

**Published:** 2017-03-14

**Authors:** Neil S. Greenspan

**Affiliations:** 1 Case Western Reserve University, Cleveland, Ohio

**Keywords:** antibody, immunoglobulin, affinity, complex formation, molecular discrimination, serum half-life, effector function, complementarity, gene rearrangement, somatic hypermutation, affinity maturation

## Abstract

In this brief commentary, I highlight the remarkable properties of antibodies (also known as immunoglobulins) revealed by more than 100 years of biomedical research. Since antibodies can be elicited through one or another means against almost any molecule or macromolecule, the universe of antibodies represents a sort of molecular mirror for the totality of molecules that make life possible. Consequently, as recounted below, antibodies play a role in almost every aspect of medicine and biomedical research.

## CENTRAL QUESTION

Over one hundred years after Emil Adolph (later von) Behring's discovery of serum factors that mediate immunity [1] earned the first Nobel Prize in Physiology or Medicine in 1901 [2], why should we celebrate antibodies, what von Behring originally called “antikorper”? Why be concerned with heavy and light chains, variable and constant domains, Fabs and Fcs, hypervariable and framework regions, isotypes, allotypes, and idiotypes? What has “antibody,” a term redolent with opposition, resounding with antagonism, resonating with subversion, come to signify?

## RANGE OF ANTIBODY FUNCTIONS

Antibodies are functionally protean proteins [3]. They serve in an unparalleled range of roles in biology and medicine by virtue of a remarkable constellation of attributes including inducibility, or potential inducibility by, and noncovalent affinity for, almost any small molecule or macromolecule, prodigious capacity for molecular discrimination, structural and functional stability, remarkably extended serum half-life, structural features conducive to detection in laboratory assays, and biologically efficacious interactions, known as effector functions. These immunity-mediating effector systems involve complement and Fc receptor-bearing cells and more recently-discovered intracellular molecules.

## GENETIC ORIGINS AND DIVERSITY

Antibodies, also known as immunoglobulins, originate in an almost unique process of gene rearrangement (the broad outlines and selected critical details of which are shared with their first cousins, the T-cell receptors) that depends on what might be referred to as a series of dynamic, approximately one-dimensional immunoglobulin gene jigsaw puzzles [4]. Each antibody polypeptide chain is constructed of 3 or 4 domains encoded by gene segments that are separated by intervening stretches of nucleotide sequence in the germline DNA and that become juxtaposed, prior to translation, by either DNA rearrangement or RNA splicing. These nucleic acid acrobatics generate new amino acid sequences, not originally encoded in the germline genome, in regions of the polypeptide chains referred to, with astonishing creativity, as variable regions. Superimposed on these mutation-generating events that are integral to the processes required for the assembly of complete antibody-encoding genes is another unique process, termed somatic hypermutation that can lead to affinity maturation (ie, increased average affinity for the antigen) [5]. The latter process, effectively localized to the genetic sequences encoding heavy and light chain variable (V) domains, further expands the repertoire of V domain amino acid sequences displayed by the population of antibody molecules. As a consequence of these multiple diversification processes, there is enormous variation of primary structure in the context of impressive conservation of tertiary and quaternary structure. For example, a single human can produce as many as 100 billion different antibodies displaying distinguishable heavy-light chain V domain pairs [6].

## COMPLEX FORMATION

The complementary relationship between variation and conservation in antibody structure foreshadows the critical functional role of another level of complementarity. Antibodies exist in large measure to form noncovalent complexes with ligands, referred to as antigens, and it is through this fundamental biochemical process that vertebrate bodily integrity is affirmed and parasitic ambition is thwarted. At least to a rough approximation, the antibody and the antigen fit together as do the complementary shapes of a three-dimensional jigsaw puzzle [7, 8]. However, intra-molecular flexibility in potentially both the antibody and the antigen, solvent effects, and other factors (eg, temperature, pressure, pH, ionic strength, and the extent of molecular crowding) external to the antibody-antigen system can influence the strength of the interaction [9]. By virtue of these features, antibody-antigen interactions serve as instructive prototypes for all noncovalent interactions in biology.

## DIVERSITY OF MOLECULAR TARGETS

Antibodies can bind to molecules in solution and on cell surfaces. They can recognize proteins (including other antibodies), peptides, polysaccharides, lipids, nucleic acids, and small organic molecules, natural or synthetic and including pharmaceutical agents. They can be elicited by and form non-covalent complexes with native, post-translationally modified, and denatured proteins. They can bind independently of one another, and they can bind via cooperative mechanisms, both inter-molecular and intra-molecular and can attach to monovalent and multivalent antigens. In addition, antibodies can sometimes engage all of their antigen-binding sites simultaneously with one bivalent or multivalent antigen, so called monogamous bivalency or multivalency, or they can crosslink independent soluble antigens or antigenic particles leading to, respectively, precipitation or agglutination. Both of these processes can be usefully exploited in research or clinical labs in addition to contributing to both physiologic and pathophysiologic mechanisms [10].

## MEDIATION OF IMMUNITY

What beneficial consequences does this capacity for forming molecular complexes bring to the producer of the antibodies? Antibodies mediate immunity by diverse means. They inactivate toxins and enzymes and perhaps antagonize other functions associated with pathogen-encoded macromolecules. They do it by denying viruses the opportunity to latch onto, penetrate, or control the biosynthetic processes of host cells. They do it by preventing bacteria, fungi, and parasites from adhering to host epithelium, and by calling in other soluble molecules, such as the complement proteins, or cell-bound molecules, the Fc receptors and their associated cells, to destroy the targets to which they have physically attached: truly guilt by association. And, they may even do it, although perhaps less frequently, by following microbes into host cells and inactivating key pathogen molecules, or by transmitting healing signals into host cells [10].

## ROLES IN PATHOGENESIS

As if this were not enough, antibodies also participate in disease pathogenesis through an equally varied range of mechanisms [10]. They can inactivate host hormones and kill, in concert with other host molecules, host cells. They can deposit in and damage tissues by eliciting the constellation of processes known as inflammation, and they can modulate inflammation as a function of their glycosylation state. They can also mimic hormones by binding to hormone receptors thereby transducing signals into cells and causing, in some cases, excessive secretion of yet other hormones, and they can decrease cell surface receptor expression, leading to failures in intercellular communication. Lastly, antibodies can contribute to the failure of almost any organ, including those newly acquired through transplantation [11].

## CONTRIBUTIONS TO DIAGNOSIS AND THERAPY

And yet there is more to elicit the envy and subvert the self-esteem of other, less versatile, molecular species. In clinical laboratories, antibodies are used to measure the concentrations in body fluids of dozens of soluble molecules, from hormones and cytokines to toxins and drugs, both legal and illegal, to detect and characterize molecules in body fluids or tissues, and to identify and enumerate normal and abnormal cell types in the blood or tissues [12]. They have been used to compute compatibility for organ and stem cell transplants [13], detoxify overdoses of therapeutic drugs [14], pinpoint paternity [15], maintain maternity [16], and as products of biotechnology, treat tumors, infections, and immune responses that damage transplanted organs [17, 18]. Coupled to radionuclides, and in conjunction with sophisticated imaging equipment, they can be used to localize antigens and cells in the living patient [19].

## CELL BIOLOGY AND SOMATIC EVOLUTION

Antibodies exist in solution and on cell surfaces. They participate in signal transduction, antigen internalization, and cellular trafficking. They traverse epithelial surfaces, going from basolateral to apical domains, sometimes even disposing of molecular refuse in the process. They naturally evolve in real time in the course of a single host-pathogen interaction [20], and they can even be “trained” (via mutation and selection, *in vivo* or *in vitro*) to carry out selected enzymatic reactions [21, 22].

## MOTIVATION FOR FOCUSING ON ANTIBODIES

So, here is an answer to the initial query: antibodies are pillars of the immune response, paragons of molecular specificity, defenders of organismal integrity, tools of diagnosis, purveyors of biotechnological therapy, and finally, as a consequence of the foregoing, emblems and icons of the immune system. If, as Richard Feynman claimed, “Nature uses only the longest threads to weave her patterns, so that each small piece of her fabric reveals the organization of the entire tapestry,” then antibodies correspond to a very long thread indeed.
